# Teaching Missing Data Methodology to Undergraduates Using a Group-Based Project Within a Six-Week Summer Program

**DOI:** 10.1080/10691898.2016.1158018

**Published:** 2016-05-09

**Authors:** Megan M. Marron, Abdus S. Wahed

**Affiliations:** 1 Center for Aging and Population Health, Department of Epidemiology, Graduate School of Public Health, University of Pittsburgh, 130 North Bellefield Avenue, Pittsburgh, PA 15213, USA.; 2 Department of Biostatistics, Graduate School of Public Health, University of Pittsburgh, 130 North Bellefield Avenue, Pittsburgh, PA 15213, USA.

**Keywords:** Method of handling missing data, Missing data mechanism, Simulation, Summer institute for training in biostatistics

## Abstract

Missing data mechanisms, methods of handling missing data, and the potential impact of missing data on study results are usually not taught until graduate school. However, the appropriate handling of missing data is fundamental to biomedical research and should be introduced earlier on in a student’s education. The Summer Institute for Training in Biostatistics (SIBS) provides practical experience to motivate trainees to pursue graduate training and biomedical research. Since 2010, SIBS Pittsburgh has demonstrated the feasibility of introducing missing data concepts to trainees in a small-group project-based setting that involves both simulation and data analysis. After learning about missing data mechanisms and statistical techniques, trainees apply what they have learned to a NIDDK/NIH-funded Hepatitis C treatment study, to examine how various hypothesized missing data patterns can affect results. A simulation is also used to examine the bias and precision of these methods under each missing data pattern. Our experience shows that under such project-based training, advanced topics, such as missing data, can be presented to trainees with limited statistical preparation, and ultimately, can further their statistical literacy and reasoning. The tools presented here are provided in the Appendix.

## 1. Introduction

The need for valid statistical analyses in medical sciences and many other fields continues to grow, exceeding the capabilities provided by the number of statisticians in the field (Bureau of Labor Statistics 2014–2015). Introducing advanced concepts in undergraduate statistics courses (e.g., Statistics 2, Mathematical Statistics, or an independent study) could possibly attract strong students to the field, or at the very least provide students familiarity with issues in data analyses. Providing a more enriching education in statistical reasoning can lead to practical statistical literacy and a greater appreciation of the topic (Hogg 1991; Holcomb and Ruffer 2000).

However, it is difficult teaching advanced statistics topics to undergraduates with a limited background in the field. The Guidelines for Assessment and Instruction in Statistics Education (GAISE) college report (Aliaga et al. 2005) provides direction on the important topic of how to construct introductory courses; these guidelines can also be used when introducing advanced topics. The recommendations focus on developing statistical literacy and statistical thinking, conceptual understanding rather than knowledge of procedures, using technology to analyze real data, active learning in the classroom, and assessments to improve and evaluate student learning. Students learn better and retain more when actively working in small groups (Keeler and Steinhorst 1995), and promoting active learning by implementing group-based projects has become a popular technique (Hogg 1991; Garfield 1993; Snee 1993; Moore 1997; Smith 1998; Holcomb and Ruffer 2000; Carver 2014).

Consistent with these recommendations, the Summer Institute for Training in Biostatistics (SIBS) at the University of Pittsburgh (Stone and Wilson 2010–2015) has successfully introduced advanced statistics topics to trainees with limited exposure to the field since 2010. Throughout the SIBS Pittsburgh program, lectures focus on the concepts rather than the mechanics behind advanced statistics methods. Trainees participate in small-group projects where they implement the methods they are learning by analyzing clinical datasets. The current report describes one such project (see the Appendix, available in the online supplemental information) in order to provide statistical educators an example and a set of tools used for teaching an advanced statistics concept to students with minimal statistics training.

Specifically, using a dataset from a multicenter Hepatitis C treatment study, SIBS trainees are taught how results differ when using different methods of handling missing data. To further enforce conceptual understanding, a simulation provided by the instructors is introduced to show how datasets containing different types of missing data can be generated, in addition to the impact each missing data mechanism has on results under each method of handling missing data. The goal of this project is to effectively teach trainees the various missing data mechanisms and methods of handling missing data. This will allow them to be more conscientious of such issues as they move forward in their careers.

## 2. Setting

### 2.1. SIBS Pittsburgh

SIBS Pittsburgh is a six-week National Heart, Lung, and Blood Institute (NHLBI) funded training program with the purpose of providing undergraduates and recent Baccalaureate graduates exposure to biostatistics and other health sciences (Stone and Wilson 2010–2015). The five main aims of SIBS Pittsburgh are to:

1. Introduce basic biostatistical methods using compelling scientific questions and real data.
2. Teach analytic and computer skills needed to address these questions.
3. Actively involve trainees in collaborative research projects.
4. Educate the role of biostatisical thinking in collaborative research.
5. Introduce educational and employment opportunities in the field of biostatistics.

The program provides trainees with a busy six weeks of lectures, labs, group project meetings, seminars, journal club, and field trips to organizations around Pittsburgh. There are six main professors at the University of Pittsburgh who lead SIBS, all are faculty in the Graduate School of Public Health with three in the biostatistics department (including the senior author in this article), two in the epidemiology department, and one in the human genetics department. Lectures are held four times a week for 2 hr, where topics such as linear regression, logistic regression, survival analysis, causal inference, and Bayesian methods, among others, are taught. Weekly seminars occur where principal investigators of research studies being conducted in Pittsburgh come to discuss the design and analysis of their study. The datasets from these studies are used in lab and in the collaborative research projects. The purpose of the seminar is to give trainees the context of the study that they will be working with, helping them become familiar so that there is more meaning to the results they obtain. There are four collaborative research projects trainees can choose from, where one to two professors mentor the group along with a couple of graduate students and/or other senior biostatistics/epidemiology mentors. Journal club also occurs weekly, where trainees get acquainted with how research articles are written and discuss the methods and results that are presented in the article. Laboratory sessions, where trainees apply what they learned in lecture to a clinical dataset using Stata 13 (StataCorp 2013), occur three times a week. Field trips to different organizations around Pittsburgh are held once a week and expose trainees to different work environments where statistics are being used.

SIBS trainees come from different colleges and universities across the nation, with a variety of different backgrounds such as mathematics, statistics, biology, psychology, and more. Trainees receive transportation to and from the University of Pittsburgh, on-campus housing, tuition for three college credits, and a food allowance of $25 per day. The only SIBS Pittsburgh requirement is having at least one semester of Calculus, thus SIBS cohorts tend to range from having no knowledge of statistics to having already taken courses such as linear regression.

### 2.2. SIBS Missing Data Project

Trainees choose to work on one of four different projects. The goal of these projects is to get trainees thinking about their own research questions or to introduce them to a new statistics topic that is not addressed during lecture. The collaborative research project discussed here uses a clinical dataset and a simulation to introduce trainees to missing data mechanisms, methods of handling missing data, and the impact of missing data on study results. Trainees explore these concepts using a multicenter treatment study of Viral Resistance to Antiviral Therapy of Chronic Hepatitis C (Virahep-C) dataset. In this study, there are 196 African Americans and 205 Caucasian Americans with hepatitis C virus (HCV) undergoing treatment of peginterferon alfa2a (180 *μ*g/week) with ribavirin (1000–1200 mg/day) for up to 48 weeks (Conjeevaram et al. 2006). Trainees are responsible for applying what they learn from project meetings to the Virahep-C study. The following two aims were evaluated in the Virahep-C study applying various methods of handling missing data in conjunction with appropriate statistical methods:

1. Estimate the mean change in HCV RNA levels between baseline and week 12 and the mean difference of the changes between African Americans and Caucasian Americans
2. Assess the associations of race, sex, weight, baseline HCVRNA level, and adherence with the changes in HCV RNA levels through a simple linear regression model.

Trainees compare results using various methods of handling missing data and explain why these similarities and differences may occur. Since the type of missing data is never truly known in a clinical dataset, the trainees are also introduced to a simulation where they are taught how to generate data under each missing data mechanism. Using the simulated datasets, each method of handling missing data was applied in order to visualize how results differ when different missing data mechanisms exist, as well as see how results compare to the model parameters.

During the last week of the program, trainees teach the rest of the trainees, who are not involved in the missing data research group, as well as the SIBS faculty, why missing data should not be ignored, how to notice differences between mechanisms, and what methods are commonly used to deal with missing data. They also present the simulation to illustrate to the class the amount of bias and variability each method produced under the different missing data mechanisms.

The missing data project differs from the other three collaborative research projects in the aspect that it is more theoretical and teaches trainees an additional topic that they can pass on to other trainees. In this way, this project directly addresses the difficulties involved in effectively teaching, in only a few lectures, an advanced statistical topic (involving related issues such as bias, precision, and efficiency) to those with no theoretical background.

## 3. Project Design

The missing data project group consists of five to six trainees who meet six times during the program for approximately 2 hr each time. The following is the layout of group meetings within the six-week program.

1. Meeting 1: Introduce missing data mechanisms and methods of handling missing data with examples;
2. Meeting 2: Introduce code to apply each method of handling missing data in Stata;
3. Meeting 3: Introduce a simulation study in statistical programming package R;
4. Meetings 4 and 5: Trainees work on project while instructors are present;
5. Meeting 6: Trainees practice their presentation while instructors are present.

### 3.1. Meeting 1: Missing Data Mechanisms and Methods

In about 2 hr, trainees are given an overview of missing data mechanisms: missing completely at random (MCAR), missing at random (MAR), and not missing at random (NMAR). In addition, trainees are provided with informative examples that make it possible to learn each mechanism in a short amount of time. Missing data mechanisms are described as assumptions about the nature of missing values. Trainees are taught the importance of dealing with and justifying missing data in a study and to always ask the question: “How did this missing data come about?” They are given a simple non-technical paper, Fitzmaurice (2008), that explains various missing data mechanisms and their implications in more detail.

Trainees are also introduced to methods of handling missing data such as complete case (CC) analysis, inverse probability weighting (IPW), and Imputation methods: last observation carried forward (LOCF), multiple regression imputation, and Monte Carlo Markov Chain (MCMC) multiple imputation. The advantages and disadvantages of each method are discussed as well as which method assumes which mechanism(s). Sections 3.1.1–3.1.3 describe missing data mechanisms and Sections 3.1.4–3.1.7 describe methods of handling missing data.

#### 3.1.1. Missing Completely at Random (MCAR)

We use the notation in Fitzmaurice (2008) to define and explain mechanisms of missing data. The outcome of interest is denoted as *Y* and the rest of the observed information as *X*. We define an indicator variable *Q* where *Q* equals 1 if *Y* is observed and 0 if it is missing. First, we explain that missing values are called missing completely at random (MCAR) if the probability of missing values has nothing to do with the observed or missing values. Then we show how it is written in mathematical notation. That is, missing values are referred to as MCAR if *P* (*Q*=0 | *Y*,*X*)=*P*(*Q*=0).

An example of MCAR describes a lab setting where due to an assay failure the researcher is no longer able to gather data for that particular day, resulting in missing values. Trainees are taught to categorize this type of missing data as MCAR, since the variable being measured has nothing to do with the assay failure.

#### 3.1.2. Missing at Random (MAR)

We explain that missing values are called MAR if the probability of missing values depends only on the observed values; using the notation defined above MAR can be written as satisfying the condition: *P* (*Q*=0 | *Y*,*X*)=*P*(*Q*=0 | *X*).

An example of MAR deals with a researcher who knows that older subjects are more likely to drop out of their study. This information can be used to predict the probability of drop out based on age, an observed measurement. Trainees learn that this type of missing data is characterized as MAR with the justification that the researcher is able to use an observed variable to predict the probability of a subject dropping out. We emphasize that MAR, which is often confused with MCAR, is different in the sense that the former depends on what has been observed, while the latter does not.

#### 3.1.3. Not Missing at Random (NMAR)

Missing values are categorized as not missing at random (NMAR) if the probability of missing values depends on the missing values themselves, and in addition it can depend on the observed values as well. For NMAR the probability of missing values cannot be simplified further as it may depend on both *Y* and *X*: *P*(*Q*=0 | *Y*,*X*). It is helpful to point out that the major difference between MAR and NMAR is that NMAR may depend on the observed data, but it has to depend somehow on the missing outcome values as well, whereas MAR only depends on the observed data.

A useful example of NMAR deals with some subjects dropping out in a weight loss study, for example, subjects who attend the initial visit, but do not return for a follow-up visit. Suppose that these subjects, prior to the follow-up visit, weigh themselves and realize they gained weight, they then assume the study is not helping them lose weight so they decide not to attend any more visits. The researcher was going to weigh these subjects at the follow-up visit, but instead has no additional information on their weight besides their initial measurement. Trainees are taught that this type of missing data is classified as NMAR since the subjects did not attend the visit because of the measurement that they would have received for their weight; thus, the probability of missing data depends on the missing data itself. However, since this information is not actually known, NMAR can never be determined with certainty. Trainees are taught that the data alone cannot distinguish between missing data mechanisms (Molenberghs and Kenward 2007). Often, exit surveys are administered after drop-out, where the information collected on the reason for drop-out is helpful in ascertaining whether the missing data mechanism is MCAR, MAR, or NMAR.

#### 3.1.4. Complete Case (CC) Analysis

**Figure 1.**
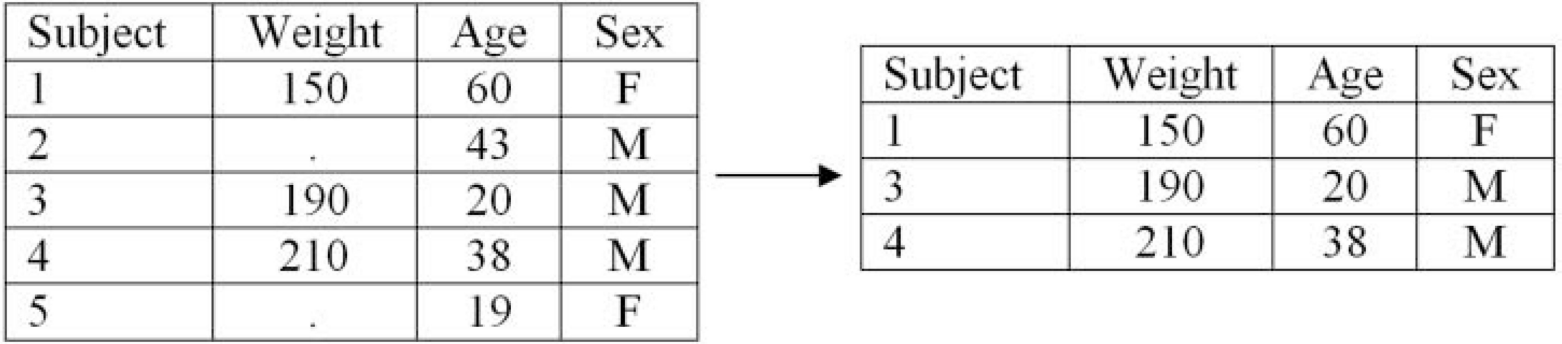
Illustration of CC analysis.

Complete case (CC) analysis is discussed first due to its simplicity. It appears easy for trainees to comprehend, especially when they learn that it is the default method of handling missing data in many different statistical software packages (e.g., Stata, SAS, R, and more) and that, unknowingly, they had been using this method of handling missing data in previous analyses conducted during lab. Figure 1 displays an example of CC analysis where there are five subjects, two of which are missing their measurement of weight (missing values are denoted throughout using a period). Trainees learn from this example that under CC analysis, a model using weight deletes subjects 2 and 5, where only subjects 1, 3, and 4 are used to draw conclusions (Figure 1, right).

Advantages of CC analysis are that it is easiest to implement. Also, if the data are MCAR, the results will be unbiased and the distribution of the observed data will not differ from the distribution of the complete data (Little and Rubin 2014). Disadvantages are that MCAR data are rare, so the majority of the time CC analysis is not a valid method of handling missing data, and even if the missing data are MCAR, one loses power and efficiency by deleting subjects from analyses.

#### 3.1.5. Inverse Probability Weighting (IPW)

The process of inverse probability weighting (IPW) is best explained through an example with a small-sample size, where trainees are easily able to calculate the results by hand. The following IPW example is made up of six subjects, where the outcome of interest is age and the observed covariates are gender and year in college (Table 1).

**Figure 2.**
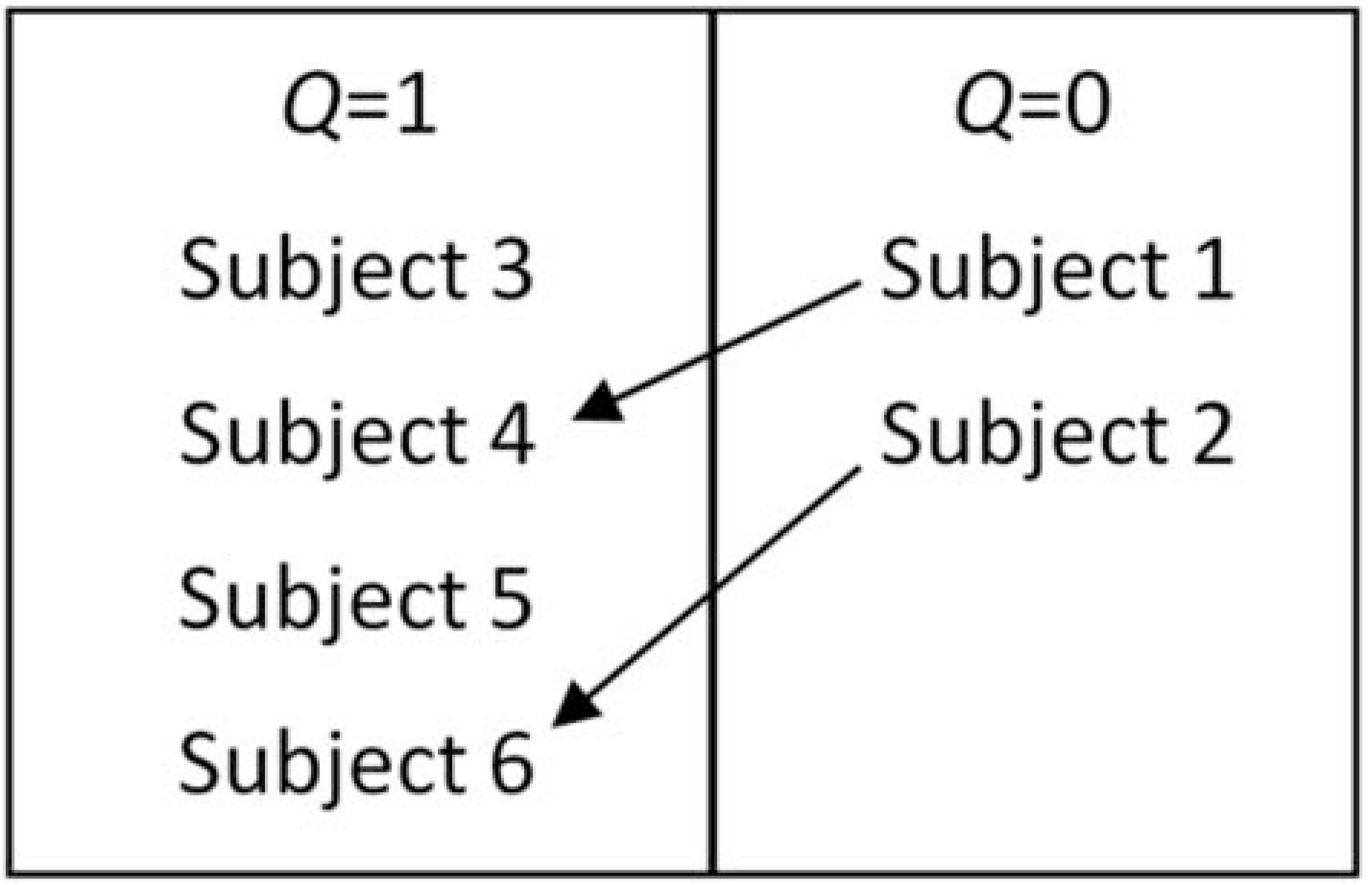
Grouping subjects based on having complete or missing data.

In order to adjust for the missing age of subjects 1 and 2 using IPW, trainees are first taught to look at the observed data to see if there are similarities between subjects who are missing the outcome of interest with those who are not. In order to illustrate this concept, each subject from Table 1 is placed into one of two boxes, depicted by Figure 2. The box is split up using indicator *Q*, where the left contains subjects with complete data and the right contains subjects who have a missing value for the outcome of interest: age. From there, trainees find similarities between subjects in the box on the right with subjects on the left. Using the observed measures (sex and year in college), the similarities found were that subjects 1 and 4 are both females who already graduated with their Bachelor’s degree and subjects 2 and 6 are both females who are in their junior year of college; therefore, subject 1 is paired with subject 4 and subject 2 is paired with subject 6. With these pairings, we show trainees that one can calculate the probability of missing the outcome of interest, age, given the observed information (*X*: sex and year in college). For example, for subject 6 (with age observed), there is one other similar individual, subject 2, whose age is unobserved. Therefore, the probability of obtaining complete information from females in their junior year of college is estimated to be ½.

After all subjects with missing values are paired off with a subject who has complete data, trainees are taught how to implement IPW in order to get an estimate of the mean age that is based on all subjects. They learn to count the subjects that have complete data as more than one individual if they are paired with another, in order to adjust for those who have missing values. In this example, the age of subject 6 is being represented twice in order to account for the missing value of age for subject 2. Similarly, the age of subject 4 is counted twice since she shares similarities with subject 1. The ages of subjects 3 and 5 are not adjusted since they did not have to account for any missing values. Using these weights, the average of the group can be calculated in a lecture setting using the following equation (Little and Rubin 2014):

$$\begin{array}{l} \text{Estimated}\;\text{Mean}\;\text{Age}=\frac{1}{6}(\text{Subject}3^{\prime }\text{sage}+2\;*\;\text{Subject}4^{\prime }\text{sage}+\text{Subject}5^{\prime }\text{sage}+2\;*\;\text{Subject}6^{\prime }\text{sage})\\ \;=\frac{1}{6}\left(\frac{Y_{\text{Subject}\;3}}{1}+\frac{Y_{\text{Subject}\;4}}{\frac{1}{2}}+\frac{Y_{\text{Subject}\;5}}{1}+\frac{Y_{\text{Subject}\;6}}{\frac{1}{2}}\right)\\ \;=\frac{1}{6}\sum_{i=1}^{6}\frac{Q_{i}Y_{i}}{\hat{\text{P}}\left(Q_{i}=1|X_{i}\right)} \end{array}$$

$\hat{\text{P}}\left(Q_{i}=1|X_{i}\right)$ indicates the estimated probability of age being observed (not missing) for a given characteristic *X*_*i*_ (sex and year in college) for the *i*th subject. The actual ages for subjects 1 and 2 can be included so that trainees can compare the average age based on complete data to the average age when missing values are present and IPW is implemented. If the true ages for subjects 1 and 2 were 26 and 20, respectively, the true average age would be estimated as 21.667. If this is taken to be the true average, the bias of the estimated average age using IPW would be 0.5 (21.667–21.167). It is also useful to have trainees figure out the mean age using CC analysis and have them determine whether the estimate using CC analysis is more or less biased than the estimate from IPW. This will help teach trainees what to look for when comparing results between different methods of handling missing data. For practical data, it is not possible to do such matches, so instead, the probability is estimated by fitting a logistic regression model, with the indicator *Q* as the outcome.

Trainees are taught that IPW assumes MAR data, which allows for calculation of the probability of complete data to be based on observed information. It is discussed that IPW differs from other methods of handling missing data since an estimate for the missing values is never produced. Instead, the observed information is used to adjust results obtained on the outcome of interest. The main advantage of IPW is that results are unbiased under MAR and MCAR (Little and Rubin 2014). Disadvantages include there is a decrease in power since it is based on the reduced sample size, and more importantly, the results will be skewed if a subject has a small predicted probability of having complete data (Seaman and White 2013).

#### 3.1.6. Last Observation Carried Forward (LOCF)

**Figure 3.**
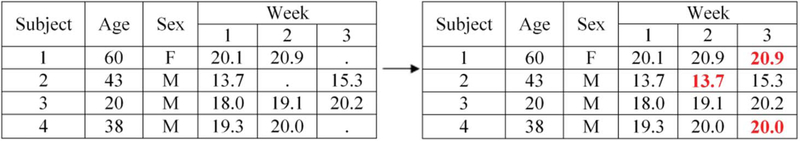
Illustration of LOCF.

Last observation carried forward (LOCF) is introduced using an example from a longitudinal study that repeatedly takes measurements on the same subjects over time. We used the Virahep-C study to illustrate an example of LOCF. The purpose of the study was to see how Caucasian Americans and African Americans respond to interferon-based antiviral therapies and whether there was a significantly different sustained virological response (SVR) between the two groups. Trainees learn that in order to see if subjects reached SVR they have to conduct a longitudinal study where multiple measurements of subjects’ HCV RNA levels are recorded over time. Once trainees comprehend a longitudinal study, the LOCF method is described as plugging in the last available measurement in place of missing values. In order to reinforce the definition of LOCF, an example, such as the one in Figure 3, was also used, where trainees filled in what the missing values would be if one were to use LOCF as their method of handling missing data.

To further reinforce the previously learned methods of handling missing data, we also asked them what they would do in the situation illustrated by Figure 3 if they wanted to use CC analysis or IPW.

Trainees are taught that LOCF might result in biased estimates even with MCAR. An advantage of the method is that it is a very simple imputation method. Disadvantages include a reduction in the sample variance by replacing missing data with identical values, which leads to conservative standard errors and increases the likelihood of Type I error (Little and Rubin 2014). LOCF is the least preferred method of handling missing data, since results become extremely biased (Kenward and Molenberghs 2009).

#### 3.1.7. Imputation

We introduce two methods of multiple imputation: regression imputation and Monte Carlo Markov chain (MCMC) multiple imputation. Regression imputation is described as imputing values for the outcome of interest when it is missing, where the imputed values are based on similarities between subjects who are missing the outcome and subjects who are not missing the outcome. The process is described below via an example. Details on regression imputation can be found in the Stata Multiple-Imputation Reference Manual (2013).

An example of regression imputation has age, sex, and race as available covariates that are significantly associated with the outcome, weight. After running a linear regression model with weight as the outcome and age, sex, and race as covariates, the age, sex, and race of the subjects who are missing a value for weight can be plugged into the regression equation to find an estimate for their weight. This process is repeated five times, where each time a different set of subjects are used to run the regression model, resulting in five complete versions of the outcome that has imputed values in place of missing measurements. Trainees are taught to run five analyses, where each analysis contains a different imputed outcome, and then average the five results obtained.

Trainees learn that regression imputation is appropriate for MAR data, where advantages include complete data on the outcome of interest with minimal bias introduced, as well as the ability to draw inference on imputed data even in the presence of a small-sample size (Little and Rubin 2014). A disadvantage is that it may be time consuming.

MCMC is described as using Markov chains based on observed information to obtain random draws from a joint distribution of variables that contain missing values. The random draws are then used to impute values in place of missing observations. Like regression imputation, MCMC imputes values for missing observations in the outcome, but it also can impute values for missing observations in predictors. Again, similar to regression imputation, MCMC is repeated at least five times, where analyses are run on the five imputed datasets and inference is made on the average of the five results. Trainees are taught that MCMC assumes at least MAR data, where advantages include being able to use more observations and being able to impute values for missing observations in the predictors, but like regression imputation it may take longer to compute (Little and Rubin 2014).

### 3.2. Meeting 2: Handling Missing Data in Stata 13

Three to five days after the first collaborative project meeting, a PowerPoint presentation on how to implement each method of handling missing data is given in a laboratory setting, where each trainee follows along with the instructor using a computer with Stata 13 (StataCorp 2013). Repetition is key when teaching advanced concepts to those with a limited statistics background, so as Stata commands for each method of handling missing data are introduced, and questions are asked to trainees based on information that they learned during the previous lecture, such as: “What missing data mechanism is assumed when using this method?,” “What are the advantages and disadvantages of this method of handling missing data?,” and so on. As trainees are taught each Stata command, they implement the command using data from the Virahep-C study. Trainees examine how results corresponding to the two aims discussed in Section 2.2 change when implementing the different methods of handling missing data.

The lecture begins by reviewing basic commands, such as how to get descriptive statistics, how to run a t-test, test of equal variances, and more. The commands for CC analysis are taught by doing nothing more than what one would do if they did not care about missing values.

When implementing IPW, trainees are taught to first create the indicator variable *Q*, where *Q*=1 if the outcome of interest is observed and 0 if it is missing. Next, they use a model building strategy to find a best-fit model for predicting *Q* (logistic regression). They then use the best-fit logistic model to calculate the predicted probability of a participant having complete data (i.e., $\hat{\text{P}}(Q=1|X))$. Last, they generate weights (the inverse of the predicted probabilities) and apply them when modeling the outcome of interest based on complete data.

**Figure 4.**
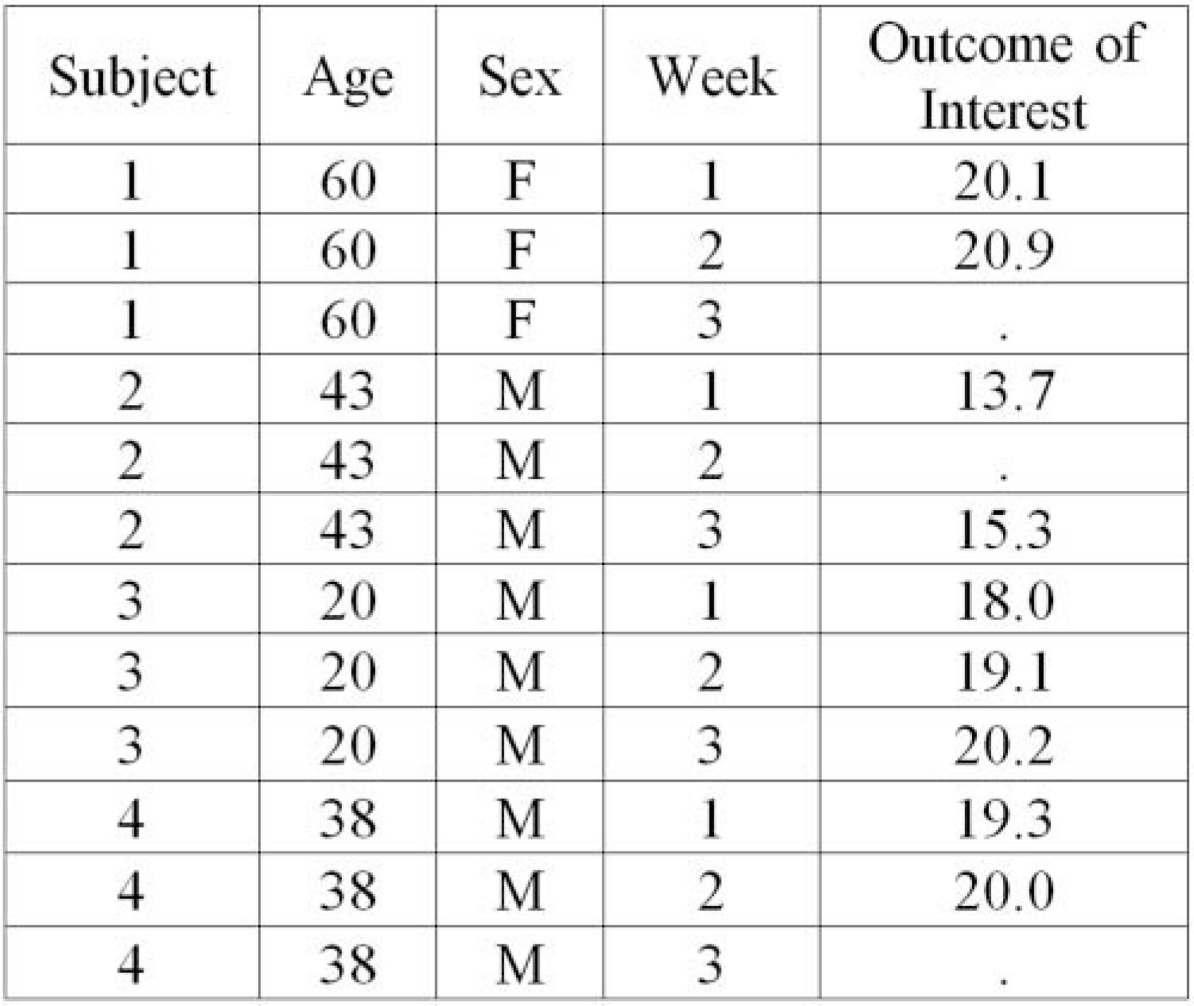
Illustration of a dataset in long format.

When introducing commands for LOCF, trainees are first taught the difference between long and wide datasets. Trainees are told that Figure 3 is an example of a dataset in wide format because each subject is taking up only a single row in the dataset and the outcome of interest that was measured repeatedly on a subject is taking up multiple columns. The trainees are then given the same dataset, but in long format (Figure 4), where the trainees see that now a single subject is taking up multiple rows, but the outcome of interest that was measured repeatedly on a subject is only taking up a single column. Next, the trainees learn how to reshape a dataset from wide to long format. Once a dataset is in long format, implementing LOCF becomes one quick line of code, where a new outcome of interest variable is generated that contains no missing values. This new variable for the outcome of interest is then used in subsequent analyses.

Regression imputation can be performed in either long or wide format. Trainees are taught to first register the outcome as the variable for which the program will impute values in place of missing observations and to specify the number of times the imputation process should be run, resulting in the same number of imputed outcomes. Last, analyses are run, each time using a different imputed outcome, and results obtained are averaged (e.g., beta coefficients, test statistics, *p*-values). MCMC is implemented very similarly to regression imputation. The distribution of the outcome is set, where missing values for the outcome of interest and any predictor variables are simultaneously replaced with imputed values. Like regression imputation, analyses are run, each time using a different set of imputed variables, and the results from each are then averaged.

While going through each method of handling missing data in Stata, trainees are taught to examine whether the sample size changed and whether the coefficients and standard errors changed and to what magnitude. Time in the lab to discuss commands took approximately 2 hr. After applying their knowledge to a clinical dataset, trainees seem to have a greater understanding of methods and the importance of properly handling missing data.

### 3.3. Meeting 3: R Simulation

Two to three days after the second group project meeting, trainees are introduced to a simulation in the statistical programming package R (R Core Team 2014) that was designed by the instructors to show the impact of ignoring missing data during data analysis. In the simulation study, samples of complete data are generated from a true population. Based off of the complete data, the simulation creates three types of datasets with missing values for the outcome, where each type of dataset contains a different missing data mechanism. The simulation then runs a linear regression model using each dataset under CC analysis, IPW, and regression imputation. The trainees are given the simulation and interpret and compare results by calculating relative bias and variance under each type of missing data mechanism when applied to each method of handling missing data. Most trainees do not have programming experience with R, thus, when introducing the simulation, the instructors also provide a short introduction to the R software package (a total of approximately 30 min long).

**Figure 5.**
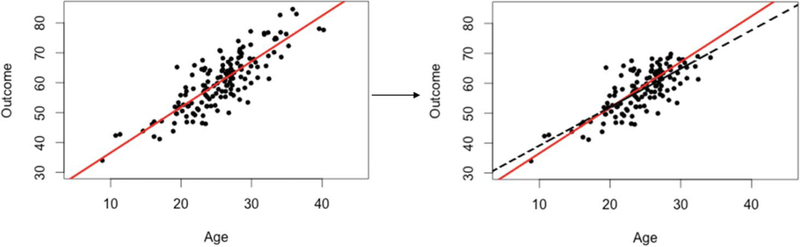
Illustrating how missing values can alter results.

The purpose of using a simulation is to illustrate the amount of bias and precision under each method of handling missing data as the missing data mechanism changes. Trainees are able to further their understanding of why missing data is problematic using a scatter plot of the simulated datasets, such as the one displayed in Figure 5. The scatterplot on the left of Figure 5 is from the simulated complete dataset with the solid line depicting the least-squares regression line. The scatterplot on the right is of the simulated dataset that contained missing values that were NMAR. The solid line on the plot is the same least-squares regression line as the one on the left of Figure 5 and the dashed line is the least-squares regression line that is obtained when doing CC analysis. Using these two plots, the trainees are able to visualize how missing data can have an effect on results.

An advantage of using a simulated dataset is that the missing data mechanism and the true values are known; thus, results gathered under each missing data mechanism and method of handling missing data can be directly compared to the true values. By calculating relative bias and variance for each method under each missing data mechanism, trainees can see which method results in the least amount of bias and/or smallest variance.

### 3.4. Meetings 4 and 5: Available Time with Instructor Present

Trainees are given two days (2 hr each) during the next week and a half to work on a group presentation while an instructor is present to answer questions. Trainees use this time to decide who will present missing data mechanisms, each method of handling missing data, and the simulation. They also use this available time to (1) finish up analyses and organize results obtained from implementing different methods of handling missing data in the Virahep-C study, (2) organize results obtained from the simulation, and (3) prepare slides for the group presentation. They are also encouraged to use their own examples to explain various missing data mechanisms and methods of handling missing data.

### 3.5. Meeting 6: Practice Presentations

During the last week (approximately three days after the last available time with instructor present) a practice presentation is held to ensure trainees demonstrate knowledge of all pertinent topics in their project. The instructors provide feedback for the trainees so that their material will be presented in a clear and concise manner. The purpose of their presentation is to teach other SIBS trainees and faculty the missing data concepts that they learned during the program. Trainees also use creative and original examples for each missing data mechanism and methods of handling missing data that the audience can appreciate. We believe it is beneficial for them to present on the concepts they learn because a helpful way to learn a topic can be to try to teach it to others.

## 4. Discussion

SIBS Pittsburgh effectively teaches advanced concepts to trainees with a limited background in statistics by following the GAISE recommendations. The SIBS project discussed here consists of introducing a small group of trainees to missing data mechanisms and methods of handling missing data through data analysis, simulation, and a presentation. The mechanics behind the methods are briefly introduced, but the focus is on understanding concepts and developing statistical reasoning and statistical thinking skills. Using examples to illustrate missing data mechanisms and methods of handling missing data is essential to trainees’ conceptual understanding. Exposure to these examples inspires trainees to come up with their own examples that involve faculty and other trainees in the SIBS program. Presenting their novel examples leads to a better understanding for both themselves and the rest of trainees since their examples are applied to a topic that everyone is familiar with (i.e., the SIBS program).

A limitation of our approach is that our trainees were motivated, passionate, and willing to learn during every project meeting. It may have been a very different experience if our group of trainees were randomly chosen from a required undergraduate level statistics course. Another limitation is that we did not collect formal assessment feedback from trainees on their experience in the group project. However, 77.8% of SIBS Pittsburgh trainees who have already graduated have either gone on to graduate school or are working in a statistics or biostatistics related job.

Instructors of an undergraduate level statistics course can use this project-based method as a template to teach their students about missing data. They can alter our technique depending on the amount of available time in their syllabus. An example of an abbreviated version is to spend a lecture teaching missing data mechanisms and briefly introducing the available methods of handling missing data. A homework assignment can then be given where students use the available simulation to compare the relative bias and variance obtained from each method of handling missing data when applied to each of the three datasets that have a different missing data mechanism. If the instructor is more interested in applying methods to a clinical dataset they can spend an additional lecture showing how to implement methods of handling missing data in a statistical package. A homework assignment or final project can then focus on students analyzing available data from the Virahep-C study by examining aims discussed in Section 2.2. Since the aims discussed involve *t*-tests and linear regression, this project can be implemented in almost any statistics course without having to introduce an additional method, other than missing data mechanisms and methods of handling missing data.

Our contribution to teaching the concept of missing data in a project-based setting is to provide educators with the tools to effectively introduce missing data mechanisms and methods of handling missing data through interactive examples and simulation. Introducing an advanced concept, such as missing data, in an undergraduate level statistics course can possibly attract strong students to the field, or, at the very least, equip students with the familiarity of advanced issues in data analysis. Doing so can provide a more enriching education in statistical reasoning that ultimately leads to practical statistical literacy and a greater appreciation of the topic (Hogg 1991; Holcomb and Ruffer 2000).

## Supplementary Material

Supplemental information

## Figures and Tables

**Table 1.** Data used to explain IPW.

Subject	Age	Sex	Year in College

1	.	F	Graduated
2	.	F	Junior
3	20	M	Junior
4	24	F	Graduated
5	21	F	Senior
6	19	F	Junior
