# Supplementary material for: Teaching Missing Data Methodology to Undergraduates Using a Group-Based Project Within a Six-Week Summer Program
Source: J Stat Educ. Author manuscript; Available in PMC 2021 Jan 25. (PMC7831542; doi:10.1080/10691898.2016.1158018)
Supplement: Supplemental information [file NIHMS1563188-supplement-Supplemental_information.docx]

**Appendix: Tools to Teach Missing Data Mechanisms and Methods of Handling Missing Data**

A1. Virahep-C Dataset

In order to obtain the Virahep-C dataset, you must register and request access from the NIDDK central repository: *https://www.niddkrepository.org/studies/virahep-c/*.

A2. Applying Methods of Handling Missing Data to the Virahep C Study using Stata 13

*Generate baseline and follow-up log Viral Levels

gen vload1 = log10(vl1)

gen vload12 = log10(vl2)

*Generate Outcome: Difference in log Viral levels at baseline and follow-up

gen diff_vload = vload12-vload1

*Examine Missing Values

mdesc

findit mvpatterns

mvpatterns

*Complete Case Analysis

sum diff_vload

sdtest diff_vload, by(race)

ttest diff_vload, by(race) unequal

regress diff_vload i.race i.SEX WGTLB vload1 adhere80

*Inverse Probability Weighting

gen q = 1

replace q = 0 if diff_vload = = .

logit q i.SEX HGTIN WGTLB i.race i.HS EMPLOY i.smokests alcwk alt ast vload1

logit q i.SEX i.race alt ast

*Now that we have our model for q, we can predict fitted values

quietly logit q i.SEX i.race alt ast

estat gof

linktest

predict pi

*Generate Inverse probabilities

gen inv_pi = 1/pi

*What is the distribution of the inverse probabilities?

hist inv_pi

regress diff_vload i.race [pweight = inv_pi]

regress diff_vload i.race i.SEX WGTLB vload1 adhere80 [pweight = inv_pi]

*LOCF

*Reshape data from wide to long

reshape long vload, i(PTID) j(time)

bysort PTID: carryforward vload, gen(vload_locf)

*Reshape data back to wide

reshape wide vload vload_locf, i(PTID) j(time)

gen diff_vload_locf = vload_locf12-vload_locf1

sdtest diff_vload_locf, by(race)

ttest diff_vload_locf, by(race)

regress diff_vload_locf i.race i.SEX WGTLB vload1 adhere80

*Multiple Imputation

mdesc diff_vload race SEX WGTLB vload1

mi set wide

mi register imputed diff_vload

mi impute regress diff_vload i.race i.SEX WGTLB vload1 adhere80, add(5) force

mi estimate: regress diff_vload i.race

mi estimate: regress diff_vload i.race i.SEX WGTLB vload1

*MCMC

mdesc diff_vload race SEX WGTLB vload1

mi set wide

mi register imputed diff_vload vload1 WGTLB

mi impute mvn diff_vload vload1 WGTLB = i.race i.SEX, add(5)

mi estimate: regress diff_vload i.race

mi estimate: regress diff_vload i.race i.SEX WGTLB vload1

A3. Simulation of Missing Data using Statistical Package R

NSim<-500 #Number of replications

n<-100 #Sample size within each replication

set.seed(4097)

#True parameters for regression model

beta1<-20

beta2<-4

beta3<-1

beta4<-1.5

sigma<-1

#parameters in vector form

beta<-c(beta1, beta2, beta3, beta4)

#Probability of Missing used to construct MCAR

pmiss<-.33

#Coefficients for model used to construct MAR

gamma1<-4

gamma2<-1/250

#Generate values for covariates: treatment, sex, and age

trt<-rbinom(n,1,.5)

sex<-rbinom(n,1,.4)

age<-rnorm(n,25,5)

#Define matrices to hold results:

cc_mcar<-matrix(ncol = 4,nrow = NSim)

cc_mar<-matrix(ncol = 4,nrow = NSim)

cc_nmar<-matrix(ncol = 4,nrow = NSim)

ipw_mcar<-matrix(ncol = 4,nrow = NSim)

ipw_mar<-matrix(ncol = 4,nrow = NSim)

ipw_nmar<-matrix(ncol = 4,nrow = NSim)

mi_mcar<-matrix(ncol = 4,nrow = NSim)

mi_mar<-matrix(ncol = 4,nrow = NSim)

mi_nmar<-matrix(ncol = 4,nrow = NSim)

#Generate data

for(i in 1:NSim){

error<-rnorm(n, 0, sigma)

#Generate outcome variable, y, based on true parameters and random error

y<-beta1+beta2*trt+beta3*sex+beta4*age+error

#MCAR data:

pmcar<-(1-pmiss)*rep(1,n)

Qmcar<-rbinom(n,1,pmcar)

ymcar<-ifelse(Qmcar,y,NA)

mcardata<-data.frame(cbind(Qmcar,ymcar,trt,sex,age))

#MAR data:

pmar<-exp(gamma1*trt-gamma2*age)/(1+exp(gamma1*trt-gamma2*(age-mean(age))))

Qmar<-rbinom(n,1,pmar)

ymar<-ifelse(Qmar,y,NA)

mardata<-data.frame(cbind(Qmar,trt,sex,age,ymar))

#NMAR:

Qnmar<-(y< = 60.5)

ynmar<-ifelse(Qnmar,y,NA)

nmardata<-data.frame(cbind(Qnmar,ynmar,trt,sex,age))

#Fit regression models (CC analysis) and save coefficients from each replication:

cc_mcar[i,]<-lm(ymcar~trt+sex+age,data = mcardata,subset = (Qmcar = = 1))$coeff

cc_mar[i,]<-lm(ymar~trt+sex+age,data = mardata,subset = (Qmar = = 1))$coeff

cc_nmar[i,]<-lm(ynmar~trt+sex+age,data = nmardata,subset = (Qnmar = = 1))$coeff

#Fit Regression models (IPW analysis):

lfit<-glm(formula = Qmcar~ trt+sex, family = binomial(logit), data = mcardata)

wmcar<-1/predict(lfit, type = “response”)

aug.mcardata<-data.frame(cbind(mcardata,wmcar))

ipw_mcar[i,]<-lm(ymcar~trt+sex+age,data = aug.mcardata,subset = (Qmcar = = 1),weights = wmcar)$coeff

lfit<-glm(formula = Qmar~ trt+sex, family = binomial(logit), data = mardata)

wmar<-1/predict(lfit, type = “response”)

aug.mardata<-data.frame(cbind(mardata,wmar))

ipw_mar[i,]<-lm(ymar~trt+sex+age,data = aug.mardata,subset = (Qmar = = 1),weights = wmar)$coeff

lfit<-glm(formula = Qnmar~ trt+sex, family = binomial(logit), data = nmardata)

wnmar<-1/predict(lfit, type = “response”)

aug.nmardata<-data.frame(cbind(nmardata,wnmar))

ipw_nmar[i,]<-lm(ynmar~trt+sex+age,data = aug.nmardata,subset = (Qnmar = = 1),weights = wnmar)$coeff

#Fit Regression models (Multiple Imputation):

mi.mcar_coeff<-matrix(ncol = 4,nrow = 5)

mi.mar_coeff<-matrix(ncol = 4,nrow = 5)

mi.nmar_coeff<-matrix(ncol = 4,nrow = 5)

reg.mcar<-lm(ymcar~trt+sex+age,data = mcardata)

reg.coeff.mcar<-reg.mcar$coefficients

resid_var.mcar<-summary(reg.mcar)$sigma**2

reg.mar<-lm(ymar~trt+sex+age,data = mardata)

reg.coeff.mar<-reg.mar$coefficients

resid_var.mar<-summary(reg.mar)$sigma**2

reg.nmar<-lm(ynmar~trt+sex+age,data = nmardata)

reg.coeff.nmar<-reg.nmar$coefficients

resid_var.nmar<-summary(reg.nmar)$sigma**2

ei<-rnorm(n,0,resid_var.mcar)

#Find the value for y based on regresion coefficients

ymcar_reg<-reg.coeff.mcar[1]+reg.coeff.mcar[2]*mcardata$trt+reg.coeff.mcar[3]*mcardata$sex+reg.coeff.mcar[4]*mcardata$age+ei

ymcar_imputed<-ifelse(mcardata$Qmcar = = 1,mcardata$ymcar,ymcar_reg)

mi_mcar[i,]<-lm(ymcar_imputed~mcardata$trt+mcardata$sex+mcardata$age)$coeff

ei<-rnorm(n,0,resid_var.mar)

ymar_reg<-reg.coeff.mar[1]+reg.coeff.mar[2]*mardata$trt+reg.coeff.mar[3]*mardata$sex+reg.coeff.mar[4]*mardata$age+ei

ymar_imputed<-ifelse(mardata$Qmar = = 1,mardata$ymar,ymar_reg)

mi_mar[i,]<-lm(ymar_imputed~mardata$trt+mardata$sex+mardata$age)$coeff

ei<-rnorm(n,0,resid_var.nmar)

ynmar_reg<-reg.coeff.nmar[1]+reg.coeff.nmar[2]*nmardata$trt+reg.coeff.nmar[3]*nmardata$sex+reg.coeff.nmar[4]*nmardata$age+ei

ynmar_imputed<-ifelse(nmardata$Qnmar = = 1,nmardata$ynmar,ynmar_reg)

mi_nmar[i,]<-lm(ynmar_imputed~nmardata$trt+nmardata$sex+nmardata$age)$coeff

}

#Bias and variance of estimators for intercept, effect of treatment, sex, and age using Complete case analysis

Rel_Bias_CC_MCAR<-100*(apply(cc_mcar,2,mean)-beta)/beta

var_CC_MCAR<-apply(cc_mcar,2,var)

Rel_Bias_CC_MAR<-100*(apply(cc_mar,2,mean)-beta)/beta

var_CC_MAR<-apply(cc_mar,2,var)

Rel_Bias_CC_NMAR<-100*(apply(cc_nmar,2,mean)-beta)/beta

var_CC_NMAR<-apply(cc_nmar,2,var)

#Bias and variance of estimators for intercept, effect of treatment, sex, and age using IPW

Rel_Bias_IPW_MCAR<-100*(apply(ipw_mcar,2,mean)-beta)/beta

var_IPW_MCAR<-apply(ipw_mcar,2,var)

Rel_Bias_IPW_MAR<-100*(apply(ipw_mar,2,mean)-beta)/beta

var_IPW_MAR<-apply(ipw_mar,2,var)

Rel_Bias_IPW_NMAR<-100*(apply(ipw_nmar,2,mean)-beta)/beta

var_IPW_NMAR<-apply(ipw_nmar,2,var)

#Bias and variance of estimators for intercept, effect of treatment, sex, and age using Multiple Imputation

Rel_Bias_MI_MCAR<-100*(apply(mi_mcar,2,mean)-beta)/beta

var_MI_MCAR<-apply(mi_mcar,2,var)

Rel_Bias_MI_MAR<-100*(apply(mi_mar,2,mean)-beta)/beta

var_MI_MAR<-apply(mi_mar,2,var)

Rel_Bias_MI_NMAR<-100*(apply(mi_nmar,2,mean)-beta)/beta

var_MI_NMAR<-apply(mi_nmar,2,var)
